# Açaí (*Euterpe oleracea Mart.*) Seed Oil and Its Nanoemulsion: Chemical Characterisation, Toxicity Evaluation, Antioxidant and Anticancer Activities

**DOI:** 10.3390/cimb46050235

**Published:** 2024-04-23

**Authors:** Katia Regina Assunção Borges, Lais Araújo Souza Wolff, Marcos Antonio Custódio Neto da Silva, Allysson Kayron de Carvalho Silva, Carmem Duarte Lima Campos, Franscristhiany Silva Souza, Amanda Mara Teles, André Álvares Marques Vale, Henrique Pascoa, Eliana Martins Lima, Eduardo Martins de Sousa, Ana Clara Silva Nunes, Rui M. Gil da Costa, Ana Isabel Faustino-Rocha, Rafael Cardoso Carvalho, Maria do Desterro Soares Brandão Nascimento

**Affiliations:** 1 Northeast Biotechnology Postgraduate Program, Renorbio, Federal University of Maranhao (UFMA), Avenida dos Portugueses, 1966 Bacanga, Saõ Luis 65080-085, Maranhao, Brazil; kareborges@gmail.com (K.R.A.B.); allysson.carvalho@discente.ufma.br (A.K.d.C.S.); 2 Adult Health Master’s Postgraduate Program—PPGSAD, Federal University of Maranhao (UFMA), Avenida dos Portugueses, 1966 Bacanga, Saõ Luis 65080-085, Maranhao, Brazil; laiswolff19@gmail.com; 3 Medicine Course, Federal University of Maranhao, Imperatriz 65915-060, Maranhao, Brazil; marcos.antonio@ufma.br; 4 Postgraduate Program in Health Sciences, Federal University of Maranhao (UFMA), Avenida dos Portugueses, 1966 Bacanga, Saõ Luis 65080-085, Maranhao, Brazil; carmemdlcampos@gmail.com (C.D.L.C.); andre_amvale@hotmail.com (A.Á.M.V.); rui.costa@ufma.br (R.M.G.d.C.); carvalho.rafael@ufma.br (R.C.C.); 5 Postgraduate Program in Biodiversity and Biotechnology of the Bionorte Network, Federal University of Maranhao (UFMA), Avenida dos Portugueses, 1966 Bacanga, Saõ Luis 65080-085, Maranhao, Brazil; 6 Professional Postgradualte Program in Animal Health Defense, State University of Maranhão, Av. Oeste Externa, 2220-São Cristóvão, São Luís 65010-120, Maranhao, Brazil; damarateles@hotmail.com; 7 Farmatec Laboratory at the Federal University of Goiás, Campus Samambaia da UFG, Goiânia 74690-631, Goiás, Brazil; henriquepascoa@ufg.br (H.P.); emlima@ufg.br (E.M.L.); 8 Graduate Program in Biosciences Applied to Health, CEUMA Universitity, São Luís 65075-120, Maranhão, Brazil; eduardo.martins@ceuma.br; 9 Coordination of the Chemical Engineering course, Center for Exact Sciences and Technology, Federal University of Maranhao (UFMA), São Luís 65080-085, Maranhão, Brazil; 10 Centre for the Research and Technology of Agro-Environmental and Biological Sciences, Universidade de Trás-os-Montes e Alto Douro, 5001-801 Vila Real, Portugal; 11 Molecular Oncology and Viral Pathology Group, Research Center of IPO Porto (CI-IPOP)/RISE@CI-IPOP (Health Research Network), Portuguese Oncology Institute of Porto (IPO Porto), Porto Comprehensive Cancer Center (Porto.CCC), 4200-072 Porto, Portugal; 12 Laboratory for Process Engineering, Environment, Biotechnology and Energy (LEPABE), Faculty of Engineering, University of Porto (FEUP), 4200-465 Porto, Portugal; 13 Associate Laboratory in Chemical Engineering (ALiCE), University of Porto (FEUP), 4200-465 Porto, Portugal; 14 Comprehensive Health Research Center (CHRC), 7006-554 Évora, Portugal; 15 Department of Zootechnics, School of Sciences and Technology, University of Évora, 7002-554 Évora, Portugal

**Keywords:** açaí oil, *Euterpe oleracea Mart.*, cervical cancer, nanoemulsion

## Abstract

This study explores a nanoemulsion formulated with açaí seed oil, known for its rich fatty acid composition and diverse biological activities. This study aimed to characterise a nanoemulsion formulated with açaí seed oil and explore its cytotoxic effects on HeLa and SiHa cervical cancer cell lines, alongside assessing its antioxidant and toxicity properties both in vitro and in vivo. Extracted from fruits sourced in Brazil, the oil underwent thorough chemical characterization using gas chromatography–mass spectrometry. The resulting nanoemulsion was prepared and evaluated for stability, particle size, and antioxidant properties. The nanoemulsion exhibited translucency, fluidity, and stability post centrifugation and temperature tests, with a droplet size of 238.37, PDI -9.59, pH 7, and turbidity 0.267. In vitro assessments on cervical cancer cell lines revealed antitumour effects, including inhibition of cell proliferation, migration, and colony formation. Toxicity tests conducted in cell cultures and female Swiss mice demonstrated no adverse effects of both açaí seed oil and nanoemulsion. Overall, açaí seed oil, particularly when formulated into a nanoemulsion, presents potential for cancer treatment due to its bioactive properties and safety profile.

## 1. Introduction

*Euterpe oleracea Mart*. (Açaí) is native to South American countries, especially in the Amazon area comprising Brazil, Venezuela, Colombia, Ecuador, Suriname and Guyana. Two regions of Brazil are important producers of açaí, namely the North (Amapá, Pará, Tocantins) and the Northeast (Maranhão) [1,2]. The species is a palm, and its fruits are spherical, globose drupe, organised in clusters comprising hundreds of individual units. These fruits are purple, pulpy, and encase a hard endocarp housing a seed with a small embryo and abundant endosperm [3,4]. 

Approximately 87% of the fruit consists of epicarp, mesocarp, and endosperm, along with cellulose (53.20%), hemicellulose (12.26%), and lignin (2.30%). It also contains total polyphenols, tannins, fibres (29.69% and 62.75%), and lipids (1.6% and 3.56%) [5]. Açaí stands out as one of the most health-beneficial fruits, with its extract, pulp, and seed brimming with various chemical compounds, including phenolics, anthocyanins (such as cyanidin-3-orutinoside and cyanidin-O-glucoside), proanthocyanidins, lignins (like aryltetrahydronaphthalene, dihydrobenzofuran, furofuran, and 8-O-4-neolignan), and tetrahydrofuran derivatives (such as epicatechin, catechin homoorientin, orientin, isovitexin, and taxifolin deoxyhexose) [6,7,8]. The oil extracted from both the pulp and seed is particularly rich in fatty acids, with 73.9% being unsaturated, comprising oleic acid (56.2%), linoleic acid (11.5%), and linolenic acid (0.8%), while saturated fatty acids constitute approximately 27.5%, including palmitic acid (24.1%) and stearic acid (1.6%). Additionally, it contains phytosteroids like β-sitospero, stimasterol, and capesterol [9,10,11].

Studies have demonstrated the pharmacological effects of açaí. Its extract has shown inhibition of weight gain, hepatic steatosis, fat and liver mass, and oxidative stress in male mice models of the C57BL strain [12]. Furthermore, the extract exhibits cholesterol inhibition [13], anti-inflammatory and immunomodulatory effects [14], treatment of cardiovascular lesions [15], cytotoxic effects in breast cancer [16,17], cardiovascular remodeling effects [18], neuroprotective roles [19], antitumour effects in prostate cancer [20], and a preventive effect in the treatment of Erlich tumours in pre-clinical studies [21].

Most studies on *Euterpe oleracea Mart.* and cancer have focused on pulp and seed extracts. Monge-Fuentes et al. (2017) demonstrated that açaí oil inhibited cell viability in B16F10 murine melanoma cell lines by 85% through apoptosis [9]. Similarly, Da Silva et al. (2023) showed that both the extract and the seed oil exhibited cytotoxic effects on colorectal adenocarcinoma cell lines CACO-2, HCT-116, and HT-29 [22].

Cervical cancer is currently the sixth leading cause of death in women worldwide. In Brazil, it is the third most common and it is estimated that 17,010 new cases will arise per year between 2023 and 2025, with an estimated risk of 15.38 cases per 100,000 women. Its geographical distribution shows that it is the second most common in the North (20.48 per 100,000) and Northeast (17.59 per 100,000), third in the Centre-West (16.66 per 100,000), fourth in the South (14.55 per 100,000) and fifth in the Southeast (12.93 per 100,000). In the state of Maranhão, by 2023, this could rise to 800 new cases of cervical cancer [23].

The potential of vegetable oils in treating cervical cancer has been explored through in vitro investigations of cervical adenocarcinoma (HeLa-HPV18) and squamous cell carcinoma (SiHa—HPV16). Joradat et al. (2020) observed that *Nepeta curviflora* oil presented antiproliferative behavior and hindered HeLa cell migration [24]. Similarly, Rezaieseresh, Shobeiri, and Kaskani (2020) assessed the cytotoxic and apoptotic effects of *Chenopodium botrys* essential oil on HeLa cell lines [25]. *Tagetes ostenii* Hicken leaf and flower oil hindered the proliferation, migration, and clonogenicity of the SiHa cell line [26], and Garcia et al. (2020) demonstrated the cytotoxic effect of *Campomanesia aureas* oil on SiHa cell lines [27].

Beyond the pharmacological properties of açaí oil, advancements in biotechnological and pharmacological potential, as well as the development of nanoemulsions, have been notable. Monge-Fuentes et al. (2017) developed a nanoemulsion of açaí oil as a photosensitiser for melanoma treatment, conducting in vitro and in vivo tests, and reported the oil as a promising source of new molecules for photosensitising therapy in melanoma treatment [9]. Additionally, Loureiro Contente et al. (2020) developed a nanoemulsion as a drug carrier model, utilising açaí oil and ketoconazole as the model drug [28]. They demonstrated açaí oil’s suitability as a vehicle for imidazole antifungals. Sanches (2023) and colleagues formulated an organogel with açaí oil and hyaluronic acid (HA), structured by 12-hydroxystearic acid (12-HSA), for topical anti-aging applications, highlighting açaí oil’s potential as a raw material for organogel development [29].

Here, we demonstrate, for the first time, the antitumour effects of açaí seed oil in cervical cancer cell lines and demonstrate the development of açaí seed oil-based nanoemulsion and its biological properties.

## 2. Materials and Methods

### 2.1. Materials

MiliQ water was obtained from the Cell Culture Laboratory (LCC) at the Federal University of Maranhão (Merck, Darmstadt, Germany) Span 80^®^ (Sorbitan monooleate), Solutol^®^ HS 15 (Polyoxyethylene 12-hydroxystearic acid), ABTS•+ [2,2′-azinobis-(3-ethylbenzothiazoline-6-sulfonic acid)], and DPPH (2,2-diphenyl-1-picrylhydrazyl) were acquired from Sigma Aldrich (São Paulo, Brazil). Açaí seed oil (AO) was extracted and analysed using analytical-grade n-hexane, methanol, sodium hydroxide, potassium hydroxide, and other analytical grade reagents from Isofar Produtos Químicos (São Paulo, Brazil). 2,5-diphenyl-3,-(4,5-dimethyl-2-thiazolyl) tetrazolium bromide (MTT), dimethyl sulfoxide (DMSO), phosphate-buffered saline (PBS), fetal bovine serum (FBS), and trypsin-EDTA solution (0.5 g porcine trypsin and 0.2 g EDTA.4Na/L balanced saline solution of Hanks), Dulbecco’s Modified Eagle Medium (DMEM), supplemented with L-glutamine (584 mg/L) and antibiotic/antimycotic (50 mg mL^−1^ gentamicin sulfate and 2 mg/L amphotericin B) were obtained from Sigma-Aldrich (São Paulo, Brazil). BD™ Cytometric Bead Array (CBA) Mouse Th1/Th2/Th17 cytokine kit and Annexin V/PI were acquired from BD Biosciences (San Jose, CA, USA). 

The cell lines, HaCat, Raw, HeLa, and SiHa, were obtained from the Rio de Janeiro Cell Bank and possessed a certificate of identity and quality (INMETRO, Rio de Janeiro, RJ, Brazil).

#### 2.1.1. Obtaining Açaí Oil

The fruits of *Euterpe oleracea Mart.* were collected in the Maracanã Ecological Park, known as the Açaí Park, located in the Maracanã neighborhood in the city of São Luís, MA, Brazil (latitude: 02°31′47″ S, longitude: 44°18′10″ W, altitude: 24 m). The fruits were collected in September. The exsiccate is stored under registration number 01425 at the Ático Seabra Herbarium (SLS) of the Federal University of Maranhão. The research was submitted to and approved by the Genetic Heritage Management System—SisGen under the code A91B0BA.

**Figure 1:**
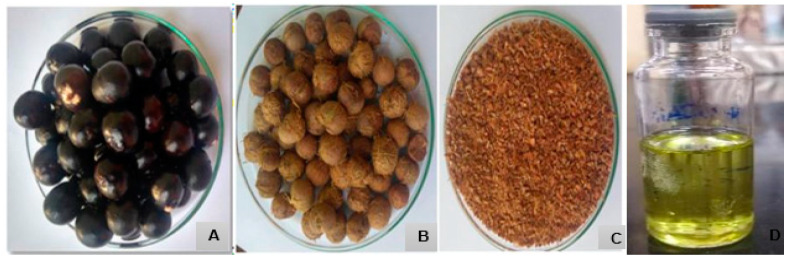
Biomass for obtaining seed oil from *Euterpe oleracea Mart.* (**A**) Whole fruit. (**B**) Seed. (**C**) Crushed seed. (**D**) Oil obtained from the seed.

The fruit collected was pulped and the seeds were exposed to the sun to dry; the fibres were removed and ground in a mill to obtain 360 g of ground seed. Extraction took place by solvent (Soxhlet) using 10 g of the crushed seeds and 300 mL of n-hexane solvent; the extraction took place at the boiling point of the solvent, and the total extraction time was 6 h (Figure 1) [21].

#### 2.1.2. Oil Analysis by Gas Chromatography Coupled with Mass Spectrometry (GC-MS)

The crude oil extracted from *Euterpe oleracea Mart.* was processed into methyl esters for GC-MS analysis, following the method outlined by Hartman and Lago (1973) [30], adapted from Lima (2007) [31]. Subsequent to processing, the fatty acids were characterised using a gas chromatograph (CG-2010) coupled with a mass spectrometer (CG-EM QP2010 Plus), both from Shimadzu (Kyoto, Japan) for chromatographic analysis, utilising a ZB-FFAP capillary column (30 m × 0.25 mm × 0.25 µm). Helium was employed as the carrier gas at a linear speed of 30 cm/s, with a column flow rate of 1.0 mL/min. The oven temperature program was set as follows: 120 °C for 2 min, with a heating ramp of 10 °C/min up to 180 °C, held for 5 min, then ramped again at 5 °C/min up to 230 °C and maintained for 25 min. The injector and ion source temperatures were maintained at 200 °C and 250 °C, respectively, with a split injection mode at a ratio of 50.

Quantification of the fatty acids was achieved by normalising the peak areas, and the esters derived from the fatty acids comprising the oil were identified using the NIST08 (National Institute of Standards and Technology) equipment library.

#### 2.1.3. Quantitative Determination of Total Phenolic Compounds and Flavonoids

The determination of total phenolics in the açaí seed oil and nanoemulsion was carried out using the Folin–Ciocalteu quantitative colourimetric method [32]. With a standard curve expressed in gallic acid (5 to 100 μg gallic acid mL^−1^). The test was carried out with 2.0 mL of 10% (*v*/*v*) Folin–Ciocalteu solution, 2.0 mL of 4% (*m*/*v*) sodium carbonate solution, and 200 µL of the 1000 µg mL^−1^ concentration of the sample (Açaí Seed Oil and Nanoemulsion); after homogenisation and protection from light for 30 min, the absorbance was read in a UV-Vis spectrophotometer (Quimis, Mod.Q-898U2M5) at 760 nm. The tests were carried out in triplicate. The total phenolic content was calculated using the regression equation obtained from the gallic acid curve.

The total flavonoid content was determined using the method described by Woisky and Salatino (1998) with adaptations [33]. For the study, 3.0 mL aliquots of the samples under study were used at a concentration of 1000 µg mL^−1^ with the addition of 300 µL of 5% ethanolic aluminium chloride solution (AlCl3), homogenised and placed under cover of light for 30 min. The absorbance was then read on a UV-Vis spectrophotometer (Quimis, Mod.Q-898U2M5) at 420 nm. The total flavonoid content was determined using a quercetin standard curve (5 to 40 µg/mL). From the straight-line equation obtained, the total flavonoid content was calculated, and the results expressed in mg of quercetin per gram of sample.

#### 2.1.4. Physicochemical Characterisation of *E. oleracea Mart*. Seed Oil

The physicochemical analyses were carried out at the UFMA Technology Pavilion, with triplicate analyses for acidity index, saponification index, and mineral material (ashes); duplicate analyses were performed for humidity, refractive index, and data obtained for density, in addition to the theoretical basis of the Adolfo Lutz Institute Standards [34].

#### 2.1.5. Obtaining a Nanoemulsion from *Euterpe oleracea Mart.* Seed Oil

##### Emulsification Method

The emulsions were prepared using the phase inversion emulsification (EIP) method according to the methodology adapted from Fernandez et al. (2004) [35] and Rodrigues et al. (2014) [36]. The aqueous (water) and oily (surfactants and açaí oil) phases were heated separately at 65 ± 5 °C. Subsequently, the aqueous phase was slowly poured under the oily phase. The system was kept under constant stirring (600 rpm) until it reached room temperature 25 ± 5 °C. After 24 h and over the course of 30 days, the formulations were subjected to stability evaluation.

##### Determination of Hydrophilic–Lipophilic Balance (HLB)

The determination of HLB was carried out based on the methodology proposed by Griffin (1949) [37], which, in this study, consisted of the preparation of nine serial formulations composed of açaí oil (*E. oleracea*) and a pair of non-ionic surfactants, both with a known HLB, A (Span 80, HLB = 4.3) and B (Kolliphor^®^ HS 15, HLB = 16), in varying proportions, giving rise to the HLB values resulting from the mixture calculated by the following equation:

Resulting HLB = (HLB A × 0.01 × A) + (HLB B × 0.01 × B)

where:

Resulting HLB = final HLB of the emulsion

HLB A = HLB of the hydrophilic surfactant;

HLB B = HLB of the lipophilic surfactant;

A = quantity used, in percentage, of surfactant A

B = quantity used, in percentage, of surfactant B

A + B = 100%

The low energy, phase inversion emulsification (PIE) method was used to obtain oil-in-water (O/W) nanoemulsions. Each formulation was prepared with a final mass of 10 g, containing 85% (*m*/*m*) distilled water, 10% (*m*/*m*) sugar oil, and 5% (*m*/*m*) of the mixture of emulsifiers A and B. To determine the HLB of the oil and choose the most stable formulation, the emulsion with the most transparent appearance and stability was considered, with no flocculation, creaming, precipitate formation, or phase separation.

### 2.2. Preliminary Stability Evaluation

#### 2.2.1. Visual Assessment

The macroscopic evaluation involved visual inspection for signs of creaming, coalescence, or phase separation (PS), along with observations of colour and appearance [38]. This assessment was conducted twice, once after 1 day and again after 30 days of preparation.

#### 2.2.2. Centrifugation Test

For stability assessment through centrifugation, 1 mL of the formulation was placed in an Eppendorf tube and centrifuged for 30 min at 15,000 rpm using a centrifuge (Eppendorf model 5415D) [39]. Following centrifugation, the sample was visually inspected. This procedure was repeated twice, once after 1 day and again after 30 days of preparation.

#### 2.2.3. Temperature Stability Test

Stability at various temperatures was assessed following the method by Sugumar et al. (2014) [40], with some modifications. The nanoemulsion was alternately stored at 4 °C and 25 °C for 24 h each. This test was conducted twice, once after 1 day and again after 30 days of preparation.

### 2.3. Physicochemical Characterisation of the Nanoemulsion

#### 2.3.1. Average Particle Size and Polydispersity Index (PDI)

The dynamic light scattering technique (Nano ZS, Malvern Instruments Ltd., Malvern, UK) was used to determine the average particle size and PDI of the formulations. Samples were analysed at 25 °C without dilution. Each sample was introduced into the cuvette of the equipment, and the Z-average was obtained from triplicate readings taken 30 days after preparation to assess storage stability. Results are presented as mean ± standard deviation.

#### 2.3.2. Zeta Potential

Zeta potential analysis was performed using a Nano ZS device (Malvern Instruments Ltd., Malvern, UK). Samples were diluted in 5 mM KCl (1:10 dilution) and analysed at 25 °C. Triplicate readings were taken for each diluted sample.

#### 2.3.3. pH Measurement

The pH of the NE-OEO was measured to evaluate potential formulation degradation. A pH meter (Hanna Instruments, Vilnius, Lietuva, HI2221-02) calibrated with standard pH 4.0 and 7.0 solutions at 25 °C ± 0.1 °C was used. Due to the fluidity of the nanoemulsion, no prior dilution was required. Triplicate analyses were conducted by directly immersing the electrode in the samples, and results were expressed as mean ± standard deviation, 30 days after preparation to assess storage stability.

#### 2.3.4. Turbidity Measurement

Turbidity analysis involved measuring the absorbance of the undiluted nanoemulsion at a wavelength of 600 nm using a Digital UV-VIS Spectrophotometer (GLOBAL TRADE TECNOLOGY, Hawthorn Woods, IL, USA). Measurements were conducted in triplicate at 25 °C, with deionised water used as a blank solution. Results were presented as mean ± standard deviation, obtained both 1 and 30 days after preparation.

In vitro biological activity of açaí seed oil and nanoemulsion based on *E. oleracea Mart*. seed oil.

Evaluation of the cytotoxicity of oil, nanoemulsion with oil (NCO) and nanoemulsion without oil (NSO) on RAW 264.7 and HaCat cells (controls) and in cervical cancer cells (HeLa and SiHa)

The RAW 264.7 macrophage cells and HaCat cells, sourced from the Rio de Janeiro Cell Bank (BCRJ), were cultured and maintained at the Genetics Laboratory of the Federal University of Maranhão—LABGEN/UFMA.

Cell viability for murine macrophage cells was determined using the MTT (thiazol tetrazolium) assay, following the protocol described by Mosmann (1983) [41]. RAW 264.7 cells (2 × 10^4^ cells/200 μL/well) were seeded into 96-well plates and incubated for 24 h in a humidified atmosphere of 5% CO_2_ at 37 °C. Subsequently, the cells were treated with varying concentrations of oil, NCO, and NSO (ranging from 0.78 to 100 μg/mL). The negative control comprised only the culture medium with cells, while the positive control involved cells treated with 10% DMSO. Following treatment, the plates were incubated in an oven at 37 °C for 24 h.

From HaCat cell line, cell viability was determined by MTT. HaCat cells (2 × 10^4^ cells/200 μL/well) were seeded into 96-well plates and incubated for 24, 48, and 72 h in a humidified atmosphere of 5% CO_2_ at 37 °C. Subsequently, the cells were treated with varying concentrations of oil (ranging from 7.8 to 1000 μg/mL).

After the incubation period, culture supernatants were removed, and the plates were washed before adding MTT reagent (Sigma Chemical Co., San Luis, MO, USA), followed by a subsequent 3 h incubation in a CO_2_ incubator, shielded from light. Following this, the plates were centrifuged at 1200 rpm for 5 min at 4 °C, the supernatant was discarded, and 100 µL of PA alcohol was added to each well. Absorbance readings were taken using a plate spectrophotometer, and the results were expressed as the percentage of cell viability relative to the controls.

Cell lines, including SiHa (HPV-16) and Hela (HPV-18), were obtained from the cell culture laboratory of the Postgraduate Master’s Program in Adult Health—PPGSAD/UFMA. These cervical cancer strains were cultured in bottles containing DMEM medium supplemented with 10% heat-inactivated fetal bovine serum (FBS), penicillin, and streptomycin, and maintained in a greenhouse with 5% CO_2_ at 37 °C.

For the treatment, SiHa (HPV-16) and Hela (HPV-18) cells were seeded into 96-well plates at a density of 1 × 10^4^ cells/mL. They were then treated with 100 µL of the oil suspension at concentrations ranging from 6.31 to 100 g/mL for tumour cell lines and 7.8 to 1000 µg/mL for normal cells. The plates were incubated for 24, 48, and 72 h in triplicate. After each incubation period, fresh medium containing 10 µL of MTT (3-(4,5-Dimethylthiazol-2-yl)-2,5-diphenyltetrazolium bromide) was added to the culture. Following a 3 h incubation in a CO_2_ oven protected from light, the plates were centrifuged at 1200× *g* rpm for 5 min at 4 °C. The supernatant was then discarded, and 100 µL of PA alcohol was added to each well for spectrophotometric readings.

### 2.4. Morphological Analysis

Morphological changes in the cells were assessed after treatment with the oil at a concentration of 50 µg/mL. An inverted microscope equipped with an Opticam O500i camera was used for this analysis. Image J (National Institutes of Health, Bethesda, MD, USA, version 1.8.0) software facilitated image analysis. Cells were cultured in 24-well plates, treated with or without the oil for 24 and 48 h, and subsequently observed and photographed under the microscope.

#### 2.4.1. Clonogenic Assay

In the clonogenic assay, SiHa (HPV-16) and Hela (HPV-18) cell lines were seeded at a density of 1.5 × 10^5^ cells/mL in a 24-well plate with 1 mL volume, incubated under a 5% CO_2_ atmosphere until reaching confluence. Following treatment with the IC50 concentration of the oil for 24 and 48 h, cells were trypsinised, and 1000 viable cells were reseeded into 12-well plates (1.5 × 10^5^ cells/mL) without treatment and incubated for 10 days. The culture medium was refreshed every 72 h. Colonies were fixed with ice-cold methanol (100%), stained with 5% Giemsa for 40 min, and observed under an inverted microscope (4×; Opticam O500i, Opticam Microscopy Technology, São Paulo, SP, Brazil). Colonies containing more than 50 cells were considered for analysis.

#### 2.4.2. Annexin-V Assay

In this cell death assay, Siha (HPV-16) and Hela (HPV-18) cell lines, cultured at a density of 1 × 10^5^ cells/mL, were treated with 50 μg/mL of açaí seed oil. Apoptosis was assessed using the Annexin V/PI staining assay to detect early, late, and live apoptotic cells. The cells were washed with ice-cold PBS and then resuspended in 100 μL of Annexin V binding buffer (0.1 M Hepes/NaOH, pH 7.4, 1.4 M NaCl, 25 mM CaCl_2_) containing Annexin V-FITC and PI (1 μg/mL) for 15 min. Subsequently, the samples were acquired using a Guava EasyCyte 8HT flow cytometer (Merck, Hayward, WI, USA) and analyzed in FlowJo v10.0 software (Treestar Inc., Ashland, OR, USA). Excitation was performed using a blue laser at 488 nm. Fluorescence detection was conducted at the Green (525/30 nm) and Red (695/50 nm) channels. Approximately 5000 events were captured per sample.

#### 2.4.3. Wound Healing Assay

For the wound healing assay, SiHa (HPV-16) and Hela (HPV-18) cell strains were cultured in 24-well plates at a density of 0.5 × 10^6^ cells/mL until reaching confluence. A linear wound was created in the monolayer using a 200 μL pipette tip, followed by three washes with PBS buffer. The culture medium containing 50 µg/mL of oil was added, and the cells were incubated at 37 °C with 5% CO_2_. After 24 h, the wound area was examined using a 40× phase-contrast microscope (Opticam O500i). The wound healing rate was determined by measuring the percentage of closure from the initial wound to complete healing at various time points, assessed by counting the cells within the scar using Image J software (version 1.8.0).

#### 2.4.4. Evaluation of Antioxidant Activity Using the ABTS and DPPH Methods

The antioxidant activity of the oil and nanoemulsion was assessed using the DPPH techniques based on the methodology of Brand-Williams and Berset (1995) [42] and ABTS as proposed by Re et al. (1999) [43] with adaptations by Teles et al. (2021) [44].

Initially, the samples were measured in triplicate, with concentrations ranging from 1000 to 250 μg/mL for both techniques (DPPH and ABTS). To carry out the measurements, 100 µL of each solution was transferred to test tubes, followed by the addition of 3000 µL of the solution of the respective radical being tested, homogenised, and kept in a dark environment for 6 min for the ABTS technique and for 30 min for the DPPH technique. Ethanol was used as a control (blank). After being kept in the dark, the absorbance was read for DPPH (517 nm) and ABTS (734), making it possible to construct the “concentration vs. absorbance” curve for each sample.

The standard curve was prepared using trolox solutions (10 to 1000 μM), in triplicate, for both techniques (ABTS and DPPH). The procedure for reading the standard curves was the same as that described for the samples, with the exception of the volume of standard used (30 µL). To calculate the concentration of 1000 μM of 8rolox corresponding to the absorbance “y” obtained, we substituted the value of “X” into the equation of the standard curve. Then, in the equations of the lines generated by the samples, we replaced the value of “y” with the absorbance corresponding to 1000 μM of 8rolox calculated. This allowed us to determine the dilution of the sample equivalent to 1000 μM of 8rolox. The “X” values found were converted into grams, and the antioxidant activity was expressed as μM 8rolox/g of sample.

The EC50 of trolox was also determined, In Ih the standard curve was Initially prepared using solutions of ABTS (50 to 1000 μM) and DPPH (50 to 1000 μM). From the linear equation obtained, the “y” value was replaced by half the initial absorbance of the control group. This made it possible to determine the corresponding amount of ABTS, expressed in μM, as described by Rufino et al. (2007) [45].

Using the straight-line equations obtained from the samples, we again replaced the “y” value with half the initial absorbance of the control group. This allowed us to determine the “X” value, which represents the Effective Concentration (EC50). The EC50 is the concentration of the sample needed to reduce the initial concentration of ABTS and ABTS by 50%.

To express it in g sample/g of ABTS or DPHH, the following equation was used:

(1) $$W=\frac{{EC}_{50}(\frac{mg}{L})/1000}{X}$$

where X is the concentration of ABTS or DPPH found by the ABTS standard curve equation, after transformation to grams.

#### 2.4.5. Evaluation of the Toxicity of the Oil and Nanoemulsion In Vivo in Swiss Female Mice

##### Animals and Ethical Aspects

The study protocol was reviewed and approved by the Ethics Committee for the Use of Animals (CEUA) at the Federal University of Maranhão, with Process No. 23115.002306/2021-66. Experimental procedures, including blood collection, were conducted at the Postgraduate Sector Bioterium within the Centre for Biological and Health Sciences (CCBS) at UFMA.

Female Swiss mice, weighing between 27 and 38 g and aged 90–120 days, were divided into five groups (8 animals per cage). They were housed under a light-dark cycle, provided with ad libitum access to food, and their weights were monitored daily for 45 days. The groups consisted of: (1) negative control (saline), (2) nanoemulsion treatment, (3) 100 mg/kg oil treatment, (4) 200 mg/mL oil treatment, and (5) 300 mg/mL oil treatment [21].

Following a 7-day acclimatisation period, the initial treatment was administered by intraperitoneal injection of 0.5 mL of saline, oil, or nanoemulsion. After 15 days of treatment, blood samples were collected from the retro-orbital region, and the animals were monitored for 4 days. A second treatment was administered following the observation period, after which the mice were euthanised using xylazine and ketamine anaesthesia (4 mg/kg bw, ip). Blood was collected via cardiac puncture, and the liver, kidney, and spleen were removed for assessment of weight, histopathology, and cellularity, respectively, using a Neubauer chamber.

##### Toxicity Assessment

The kidney and liver samples underwent fixation in 10% formaldehyde buffered at pH = 7.0 and were subsequently embedded in paraffin blocks. Histological sections of 5 µm thickness were obtained, stained with hematoxylin and eosin, and analysed using an optical microscope.

For the liver, the following parameters were assessed:

- Hepatocyte vacuolar degeneration, categorised as mild if restricted to the centrilobular zone or multifocal, and moderate if panlobular or diffuse throughout the organ.
- Presence of hepatitis, graded as mild if confined to the portal or centrilobular spaces, and moderate/severe if characterised by the formation of leukocyte bridges between portal spaces and centrilobular veins or areas of fibrosis.
- Identification of areas of necrosis, described as liquefaction or coagulation necrosis.
- Evaluation of karyomegaly, indicating hepatocytes with nuclei twice the normal diameter and exhibiting a pale and rarefied chromatin pattern.
- Counting of mitotic figures as a sign of cell regeneration, assessed per high magnification field.

In kidney analysis, the parameters included:

- Tubular degeneration, defined by the swelling of cells in the proximal or distal convoluted tubules, necrosis of isolated tubular cells, or loss of cellular vesicles into the tubular lumen (blebbing).

### 2.5. Evaluation of the Immunological and Inflammatory Response

For this study, two blood samples were collected: the first from the retro-orbital region after 15 days of treatment and the second after the second treatment, coinciding with the euthanasia of the animals. Cytokine levels were determined by flow cytometry using the Mouse Th1/Th2/Th17 Cytometric Bead Array kit (BD Biosciences, San Jose, CA, USA), following the manufacturer’s instructions. The quantification of cytokines included interleukin-2 (IL-2), interleukin-4 (IL-4), interleukin-6 (IL-6), interferon-γ (IFN-γ), tumour necrosis factor (TNF), interleukin-17A (IL-17A), and interleukin-10 (IL-10). Samples were acquired using a FACSCalibur flow cytometer (BD Biosciences, San Jose, USA) and analyzed with FCAP Array v3.0 Software (BD Biosciences, San Jose, CA, USA). 

### 2.6. Statistical Analysis

Data were presented as means ± standard error of the means. Differences were analysed using analysis of variance (one-way or two-way ANOVA), followed by Dunnett’s and Tukey’s post-tests, using GraphPad Prism v9.0 software (GraphPad SoftwareInc.; San Diego, CA, USA). The confidence interval was set at 95%, and significance was considered at *p* < 0.05.

## 3. Results

### 3.1. Chromatographic Profile of E. oleracea Mart. Seed Oil

The oil extracted from the seeds of *E. oleracea Mart* showed a yield of between 5% and 6% in relation to the dry weight of the seeds, with a characteristic olive green colour.

A detailed analysis, conducted using gas chromatography coupled with mass spectrometry (GC-MS), provided valuable insights into the chemical composition of the plant in question. During this analysis, 17 distinct chromatographic peaks were identified and quantified, corresponding to the main volatile components of the essential oil.

**Figure 2:**
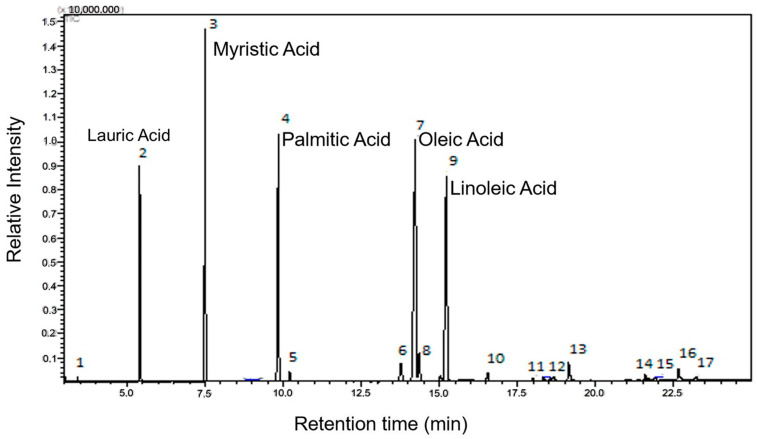
Chromatogram of the esters derived from the fatty acids in the seed oil of *Euterpe oleracea Mart*.

Of particular relevance, it was observed that this oil has a division balanced between saturated fatty acids (representing 49.27% of the total) and unsaturated fatty acids (which comprise the remaining 50.73%). Among the unsaturated fatty acids, 29.73% are monounsaturated, while 20.85% are polyunsaturated (as shown in Figure 2). This chemical composition is of great importance as it directly influences the properties and potential uses of the essential oil of *E. oleracea Mart.* (Figure 2).

Analysis by gas chromatography coupled with mass spectrometry (GC-MS) enabled the identification and quantification of methyl esters of lauric (C12:0), myristic (C14:0), palmitic (C16:0), and linoleic (C18:2), oleic (C18:1) fatty acids, with oleic acid having the highest percentage. However, other esters in smaller quantities were detected from capric (C10:0), palmitoleic (C17:1), linolenic (C18:3), stearic (C18:0), eicosanoic (C20:0), behenic (C22:0), and tricosanoic (C23:0) fatty acids. Unesterified myristic and palmitic acid and benzoic and azelaidic fatty acids were also detected, corresponding to 0.19%.

### 3.2. Chemical Quantification of E. oleracea Mart Seed Oil and Nanoemulsion

The content of total phenolic compounds found in the açaí oil and nanoemulsion, as shown in Table 1, was calculated using the regression equation y = 0.0009x − 0.0681, with an R2 of (0.9961). The total flavonols content was calculated using the regression equation y = 0.028x − 0.0625, with an R2 of (0.9938).

The content of total phenolics in the oil was 127.40 ± 0.449 mg of gallic acid equivalent per gram (mg EAG g^−1^), while in the nanoemulsion, this value was significantly higher, reaching 146.00 ± 0.259 mg EAG g^−1^. With regard to total flavonoids, the oil showed a content of 62.62 ± 0.930 mg of quercetin equivalent per gram (mg EQ g^−1^), while the nanoemulsion showed an even higher value, reaching 113.80 ± 0.454 mg EQ g^−1^.

These results indicate that the açaí nanoemulsion has a higher concentration of total phenolic compounds and total flavonoids compared to the oil, suggesting potential superior antioxidant and nutritional benefits in the nanoemulsion.

### 3.3. Physicochemical Characterisation of E. oleracea Mart. Seed Oil

Table 2 describes *E. oleracea Mart*. seed oil characteristics, regarding acidity, humidity, saponification index, refractive index, incineration residue (ash), and density. Some values were higher than normal due to the degree of purity and the time taken to extract the oil.

### 3.4. Determining the Hydrophilic–Lipophilic Balance (HLB) of Euterpe oleracea Mart. Oil and Formulating Its Nanoemulsion

Table 3 illustrates the results obtained from nine formulations prepared by blending a hydrophobic surfactant (Span 80, HLB = 4.3) with a hydrophilic surfactant (Kolliphor^®^ HS 15, HLB = 16), yielding HLB values ranging from 4.3 to 16. Upon assessment after 1 and 30 days of preparation, only formulation 5 exhibited a stable, bluish translucent appearance, devoid of any signs of flocculation, creaming, precipitate formation, or phase separation. The remaining formulations were discarded.

The calculated HLB value of the açaí oil coincided with the outcome observed in formulation 5, registering at 10.15. This particular formulation comprised 5% surfactants (50% Span 80 and 50% Solutol) and 10% açaí oil, constituting the oil phase, along with 85% PBS, constituting the aqueous phase. The resulting 10 g formulation yielded a stable oil-in-water (O/A) nanoemulsion, characterised by the desired fluidity, bluish tint, and translucency, thus selected for subsequent experiments in this investigation.

### 3.5. Stability Assessment

The selected açaí oil nanoemulsion (NE-OEO) was evaluated for stability in relation to macroscopic analysis, centrifugation test and the effect of room temperature (25 °C) and refrigeration (4 °C). The physical stability of the ideal nanoemulsion, which had a bluish translucent visual appearance, remained unchanged during all the tests, with no cream formation, sedimentation, or phase separation after 1 and 30 days of preparation.

### 3.6. Physicochemical Characterisation of the Formulation

**Figure 3:**
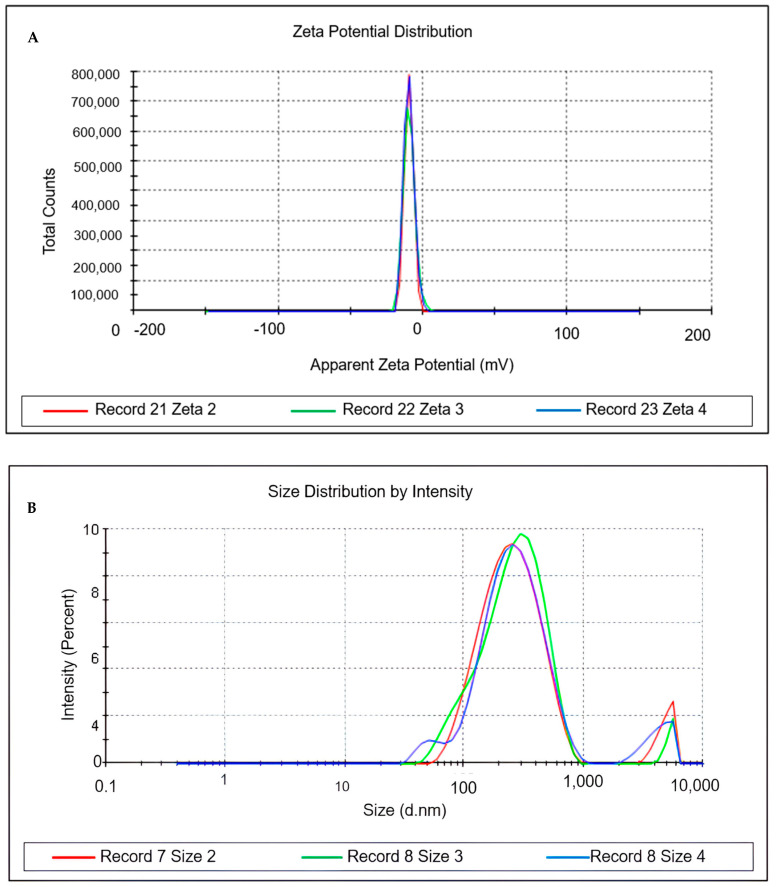
Characterisation of particle size, polydispersity index and Zeta potential of nanoemulsion with *Euterpe oleracea Mart.* seed oil. (**A**) = Zeta potential. (**B**) = Size distribution by intensity.

The ideal nanoemulsion selected was characterised 30 days after preparation in terms of droplet size, homogeneity (PDI), zeta potential, pH, and turbidity, as shown in Table 4 and Figure 3.

The formulation was nanometric in size, with a PDI indicating monodisperse particles and a negative zeta potential. It also had a neutral pH and low turbidity, characteristic of a translucent nanoemulsion.

### 3.7. In Vitro Biological Activity of Açaí Seed Oil and Nanoemulsion Based on Açaí Seed Oil

#### 3.7.1. Antioxidant Activity

The results of the antioxidant activity of the açaí oil and nanoemulsion were determined using the DPPH and ABTS tests, and were compared with a synthetic antioxidant (trolox). The values obtained are shown in Table 5, expressed as Trolox equivalents.

In the DPPH test, the essential oil showed an average antioxidant activity of 5.993 µM ET/g, while the nanoemulsion exhibited superior antioxidant activity, with an average of 9.993 µM ET/g. This suggests that the Eo seed nanoemulsion has a more robust antioxidant capacity compared to the essential oil. In the ABTS test, the results show that the essential oil had an average antioxidant activity of 6.567 µM ET/g, while the nanoemulsion obtained a significantly higher average value of 11.9 µM ET/g. Again, this indicates that the açaí seed nanoemulsion has a higher antioxidant capacity than the oil. 

Table 6 shows the results of the antioxidant activity expressed as 50% effective concentration (EC50) in the açaí oils and açaí oil nanoemulsions, using the DPPH and ABTS methods, compared to Trolox as the reference antioxidant.

**Figure 4:**
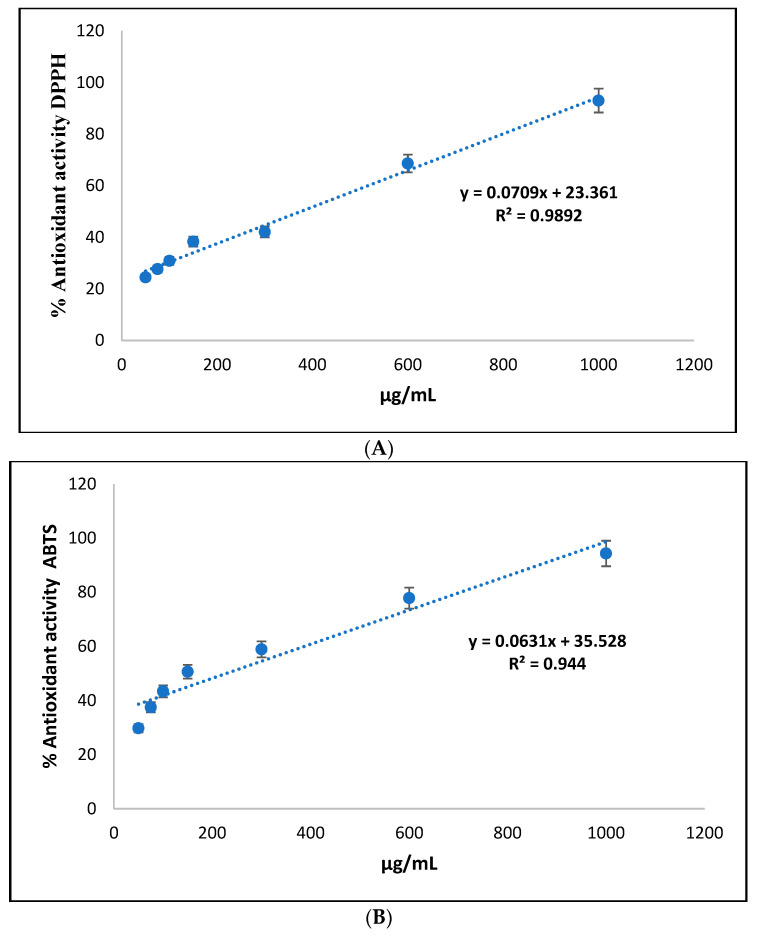
Percentage curve of DPPH and ABTS radical inhibition against *Euterpe oleracea* Mart seed oil. In the DPPH test, the EC50 of Eo oil was calculated at 375.698 µg/mL, while açaí oil nanoemulsions showed a significantly lower EC50 value, equivalent to 229.845 µg/mL. Trolox, on the other hand, showed an even lower EC50, indicating its high antioxidant capacity compared to the natural samples (**A**). In the ABTS test, the EC50 of the açaí oil was 272.0208 µg/mL, while the açaí oil nanoemulsions showed an even lower EC50 value, measuring 201.2895 µg/mL. Trolox again showed a significantly lower EC50 compared to the natural samples (**B**).

In the DPPH test, the EC50 of Eo oil was calculated at 375.698 µg/mL, while açaí oil nanoemulsions showed a significantly lower EC50 value, equivalent to 229.845 µg/mL. Trolox, on the other hand, showed an even lower EC50, indicating its high antioxidant capacity compared to the natural samples (Figure 4A). In the ABTS test, the EC50 of the açaí oil was 272.0208 µg/mL, while the açaí oil nanoemulsions showed an even lower EC50 value, measuring 201.2895 µg/mL. Trolox again showed a significantly lower EC50 compared to the natural samples (Figure 4B).

#### 3.7.2. Antitumour Activity of the Oil

Effect of *Euterpe oleracea Mart.* seed oil on the viability of queratinocites.

**Figure 5:**
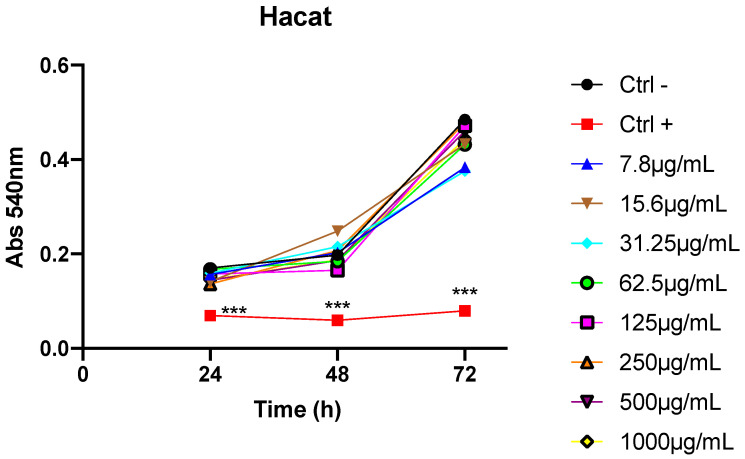
Effect of *Euterpe oleracea Mart.* seed oil on the viability of cervical cancer cell lines. *** *p* < 0.001.

To evaluate the oil’s toxicity against queratinocite cell lines, the MTT cell viability assay was conducted on HaCat cell line, at concentrations ranging from 7.8 to 1000 µg/mL. Açaí seed oil induced proliferation in these cells with no toxicity even at the highest concentration (Figure 5).

**Figure 6:**
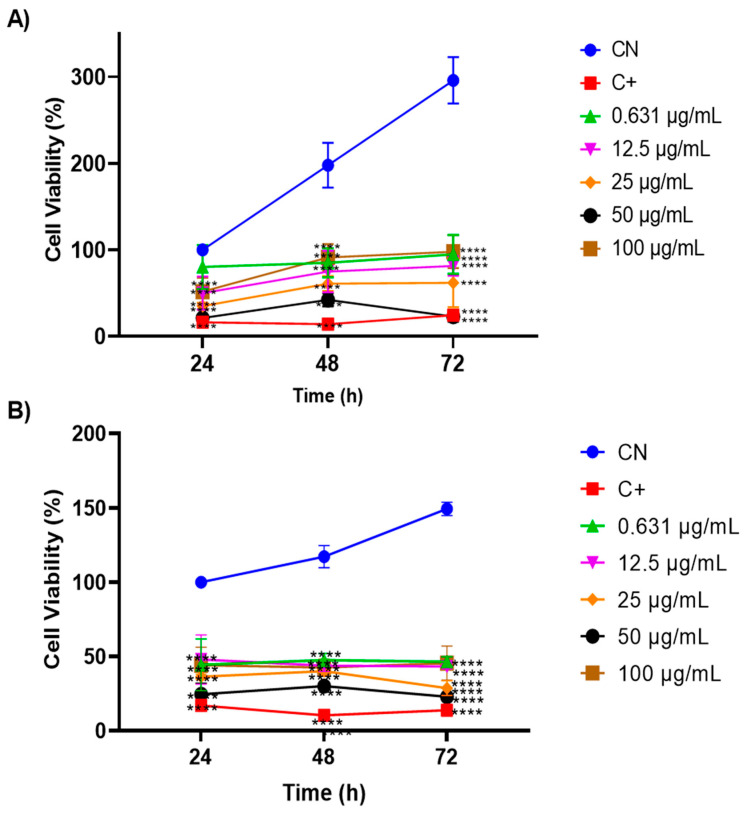
Evaluation of the antitumour activity of *Euterpe oleracea Mart.* oil in cervical adenocarcinoma cell lines: (**A**) HeLa and (**B**) SiHa using the MTT viability assay. One-way ANOVA, Tukey’s post-test. **** *p* < 0.001.

To evaluate the oil’s anti-tumour potential, the MTT cell viability assay was conducted on two cervical-adenocarcinoma-derived cell lines, HeLa and SiHa, at concentrations ranging from 0.631 to 100 µg/mL (Figure 6). Both the HeLa (Figure 6A) and SiHa (Figure 6B) cell lines exhibited significant growth inhibition (*p* < 0.001) with similar trends at concentrations of 25 and 50 µg/mL over 24, 48, and 72 h. Notably, the 50 µg/mL concentration consistently demonstrated superior efficacy, resembling the positive control profile. Moreover, the findings suggest compound selectivity, as concentrations of 0.631, 12.5, and 100 µg/mL displayed minimal anti-proliferative effects.

### 3.8. Açaí Seed Oil Induced Apoptosis in Cervical Cancer Cell Lines

Cell death mechanisms were assessed using the Annexin V-FITC/propidium iodide (PI) assay, which are two markers for differentiating cell apoptosis and necrosis. Where cells in early apoptosis are labelled with Annexin V (Annexin V+/PI), cells in late apoptosis are labelled with both Annexin V and PI, and cells in necrosis are labelled PI (Annexin V−/PI+). 

**Figure 7:**
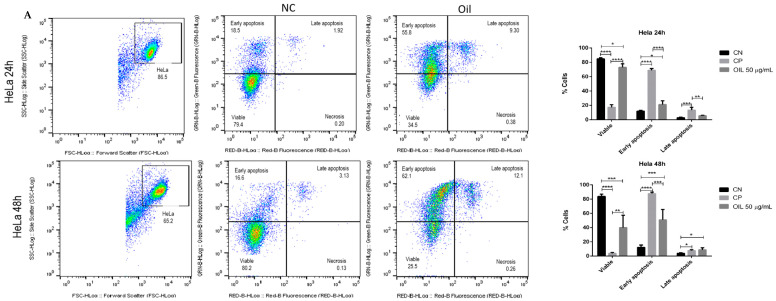

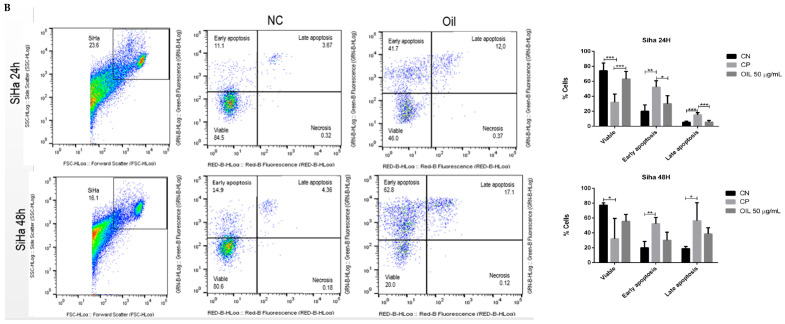
Evaluation of cell death induced by açaí seed oil (50 µg/mL) in cervical cancer cell lines (24 and 48 h) HeLa and SiHa. * *p* < 0.05, **, *p* < 0.01 *** *p* < 0.001, **** *p* < 0.0001 (**A**) HeLa, (**B**) Siha.

To assess the mechanism of death induced by *E. oleracea* seed oil, HeLa and Siha cells were incubated with 50 µg/mL of oil for 24 and 48 h, and then annexin and PI were labelled. The data illustrated in Figure 7 show that the oil (50 µg/mL) decreased the number of viable cells compared to the negative control. The data show that in the 24 h and 48 h periods, initial apoptosis occurred significantly, with 55.8% in HeLa (Figure 7A) and 41.7% in Siha (Figure 7B) at 24 h, and 62.1% in HeLa (Figure 7A) and 68.8% in Siha (Figure 7B) at 48 h. Although late apoptosis was low, the results were significant (*p* < 0.05), with HeLa at 9.3% (Figure 7A) and Siha at 17.1% (Figure 7B).

### 3.9. Açaí Seed Oil Induced Morphological Chances in Cervical Cancer Cell Lines

**Figure 8:**
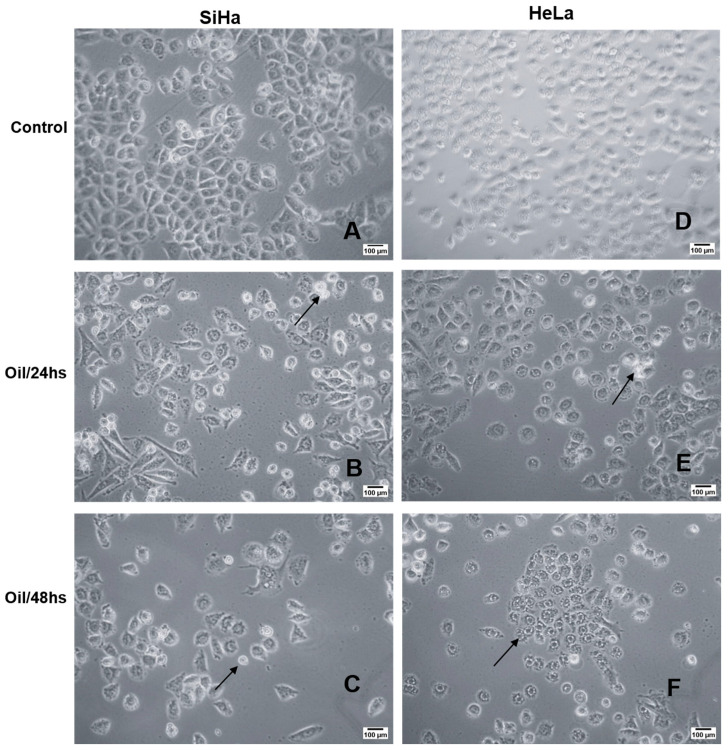
Morphological analysis by light microscopy of the cytotoxic effects of *E. oleracea* seed oil on Siha (**A**–**C**) and HeLa (**D**–**F**) cell lines. *E. oleracea* seed oil induced morphological changes in both cells; from 24 h onwards, rounding was observed (arrows).

The morphology of SiHa and HeLa cell lines, upon treatment with *E. oleracea* Mart seed oil, was examined using an inverted phase-contrast microscope, and their surface area and perimeter were analysed using ImageJ software. Control cells exhibited typical morphology characterised by elongated cells, monolayer growth, and cell-to-cell adhesion (Figure 8A,B). Upon treatment with the IC50 concentration of the oil for 24 and 48 h, both cell lines displayed changes in shape, including the formation of a distinct circular area around the nucleus and a reduction in size (Figure 8C–F). Treated cells exhibited an increase in surface area and perimeter compared to the untreated cells (Table 7). Specifically, untreated Hela cells (control) had an area of 53.8 ± 30.0 µm^2^ and a perimeter of 28.0 ± 9.9 µm^2^, while Hela cells treated for 24 and 48 h showed an area of 430.6 ± 14.4 µm^2^, 60.6 ± 19.7 µm^2^, and a perimeter of 72.7 ± 11.9 µm^2^, 27.4 ± 4.7 µm^2^, respectively. Similarly, untreated SiHa cells (control) had an area of 72.2 ± 16.5 µm^2^ and a perimeter of 33.7 ± 5.9 µm^2^, whereas SiHa cells treated for 24 and 48 h exhibited an area of 220.6 ± 89.24 µm^2^, 60.35 ± 29.9 µm^2^, and a perimeter of 57.57 ± 19.96 µm^2^, 27.0 ± 6.2 µm^2^, respectively. Compared to the control, treated cells displayed altered size and a rugged appearance on the cell surface, suggesting a potential loss of cytoplasmic material.

### 3.10. Açaí Seed Oil Exerts Effects on Clonogenic Capacity of Cervical Cancer Cell Lines

**Figure 9:**
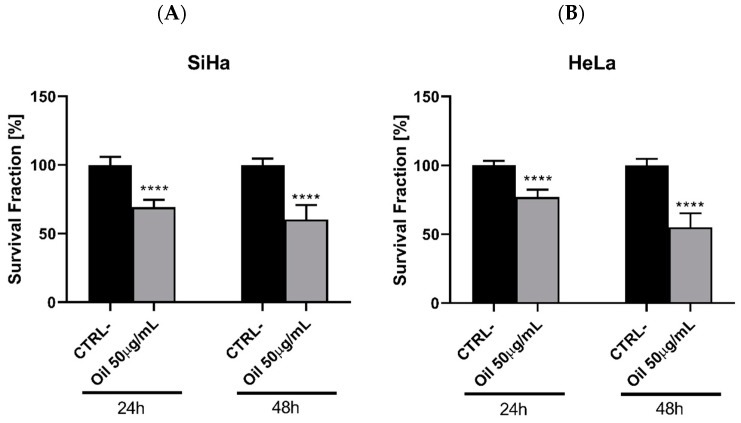
Clonogenic assay illustrates that the oil at 50 µg/mL exerted a significant inhibitory effect (*p* < 0.001) on colony formation in both SiHa (**A**) and Hela (**B**) strains compared to the control **** *p* < 0.0001. The statistical test applied was Student’s *t*-test.

The colony formation assay assesses the cells’ capability to survive and proliferate under treatment, offering insights into tumour progression and the efficacy of oncological treatments [46]. Here, we examined the impact of the oil on cell viability reduction, colony formation, and colony persistence post-treatment withdrawal using SiHa (Figure 9A) and Hela (Figure 9B) strains treated with 50 µg/mL oil for 24–38 h.

Figure 9 illustrates the clonogenic assay analysing the ability of the SiHa and HeLa strains to form colonies after being treated with the 50 µg/mL concentration of oil and being removed after 24 h and 48 h. This suggests a decrease in the number of cells per clone over time, indicating a potential anti-proliferative effect of the oil.

### 3.11. Açaí Seed Oil Decreases Invasion and Migration of Cervical Cancer Cell Lines

Cell migration can be quantified using the average distance of the groove width between its edges. To do this, ImageJ software (NIH, Bethesda, MD, USA) was used, manually measuring the images taken at 0 h and after 24 and 48 h of treatment. The lesion area was determined in millimetres (mm).

**Figure 10:**
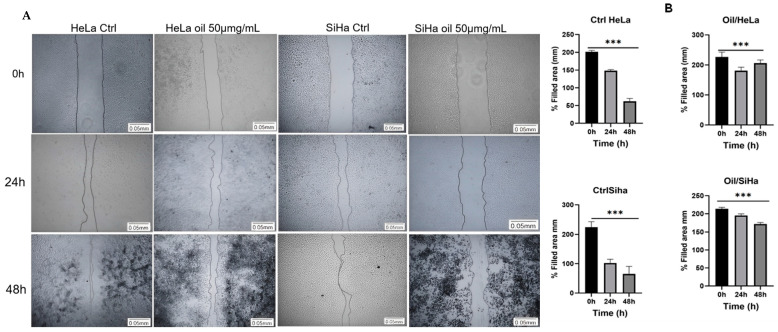
*E. oleracea* seed oil regulates invasion and migration of cervical cancer cells. Analysis of cell migration in HeLa and SiHa (**A**,**B**), *** *p* < 0.001.

After 24 h and 48 h of scarring, the wound healing assay showed that untreated HeLa and SiHa cells (Ctrl) were able to cover ≅ 70% and 99% of the wound in 48 h. The average cell migration was 126 cells for HeLa in 24 h and 181 cells for SiHa, while the average cell migration in 48 h was >1000 for HeLa (*p* > 0.001, n = 15, Figure 10A,B) and 761 cells (*p* < 0.001, n = 15, Figure 10A,B) for SiHa. In the case of the oil-treated cells, the number of migrating cells was reduced, and the wound did not close. Only 47 cells were counted in 24 h in HeLa and 71 in SiHa in the scar area, while at 48 h the average was 18 cells counted in Hela and 9 in SiHa (*p* > 0.001, n = 15, Figure 10A,B).

Figure 10B shows that there was not enough cell migration to close the scar in the treated cells (Oil) at 24 and 48 h, showing similarity with the area (mm) at time 0 h for both cells. In untreated cells (Ctrl), cell migration towards the scar reduced the size of the scar area (mm).

### 3.12. Toxixity Analysis In Vitro and In Vivo

#### Cytotoxicity Assessment with RAW 264.7 Cells

**Figure 11:**
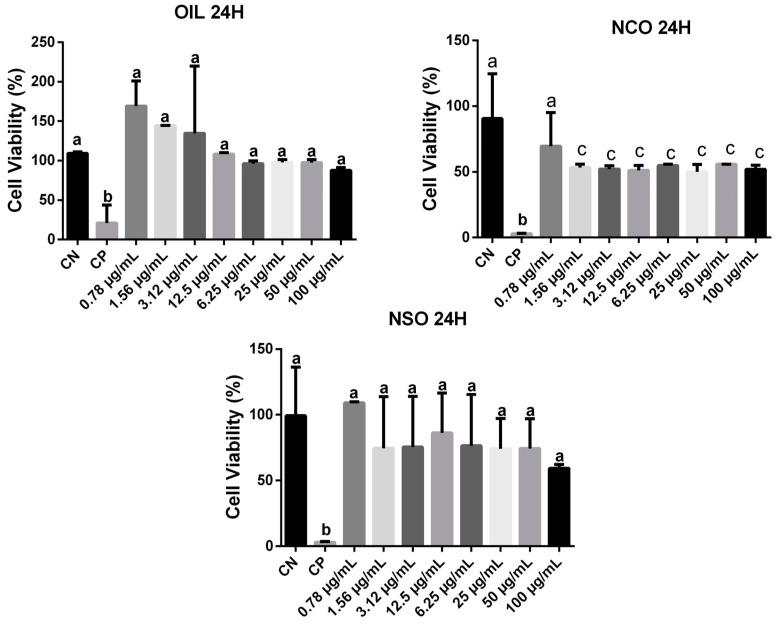
Cytotoxicity in RAW 264.7 cells treated with açaí seed oil (OIL), oil nanoemulsion (NCO) and oil-free nanoemulsion (NSO). The results were expressed as mean ± SD (n = 6 tests) and analyzes were carried out using the one-way Anova test and Tukey post-test (*p*-value < 0.05 being significant). For treatment with OIL for 24 hours (OIL24H), it was observed that the negative control was statistically equivalent to the oil concentrations. The symbol ‘b’ indicates that the positive control differed significantly from the concentrations tested. In the case of treatment with NCO for 24 hours (NCO 24H), the symbol ‘a’ indicates that the negative control was statistically equivalent only to concentrations of 0.78 μg/mL. While the symbol ‘b’ indicates that the positive control was statistically different from both the negative control and the concentrations under study and the symbol “c” indicates that was no statistically difference among NCO concentrations testes. For treatment with NSO for 24 hours (NSO 24H), the symbol ‘a’ indicates that the negative control was statistically different from the positive control and equivalent to the concentrations tested.

The MTT test was used to check cytotoxicity in RAW 264.7 cells treated with açaí seed oil (OIL), oil nanoemulsion (NCO), and oil-free nanoemulsion (NSO) (Figure 11). The oil and oil-free nanoemulsion were not cytotoxic to Raw 264.7 cells.

In vivo biological activity of açaí seed oil and nanoemulsion based in *E. oleracea* seed oil.

Açaí seed oil and açaí nanoemulsion did not significantly reduce the body weight or organ weights of mice.

**Figure 12:**
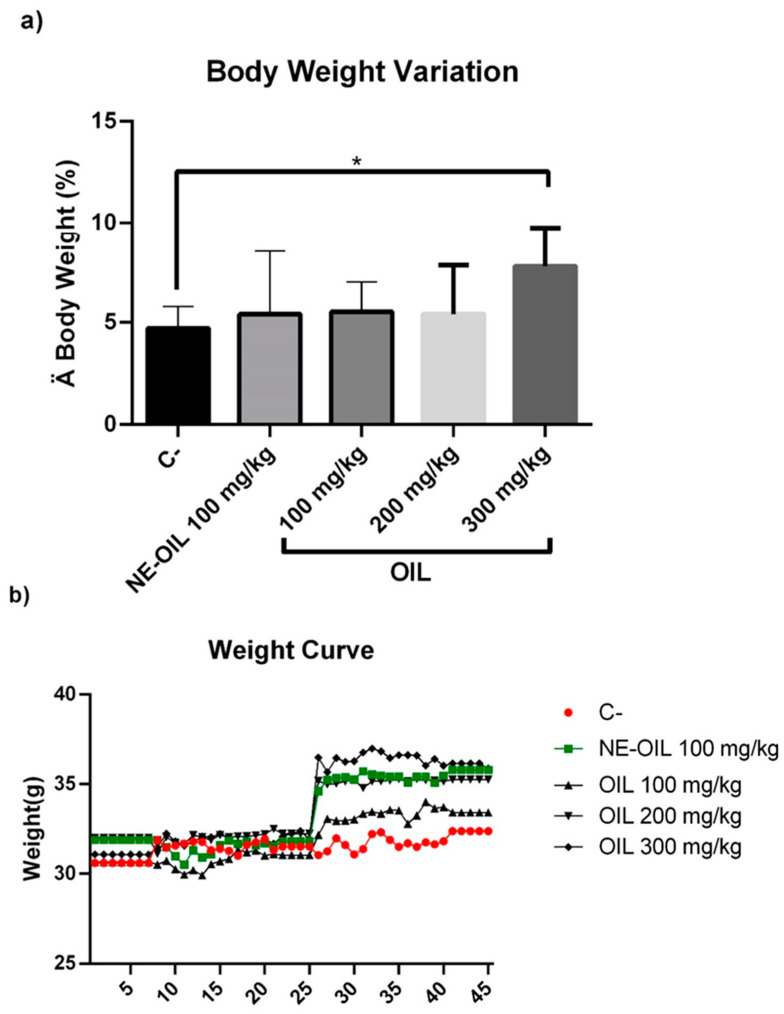
Effect of 100, 200 and 300 mg/kg oil and nanoemulsion (NE-OEO) on the animals’ weight variation at the end of treatment (**a**) and on weight kinetics throughout treatment (**b**). Statistical analysis was carried out using a one-way ANOVA test, followed by Tukey’s test for multiple comparisons. Asterisks (*) indicate statistically significant differences with a value of *p* < 0.05.

**Figure 13:**
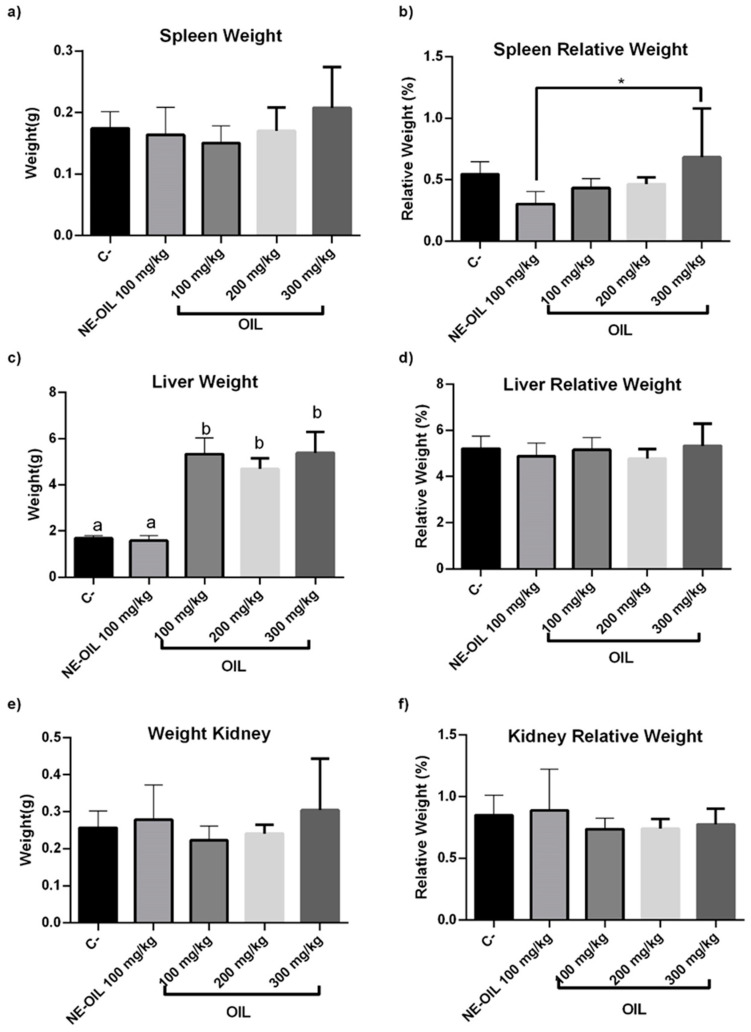
Effect of 100, 200 and 300 mg/kg oil and nanoemulsion (NE-OEO) on weight variation in animals at the end of treatment. The analysis of the absolute and relative organ weights, including the spleen (**a**,**b**), liver (**c**,**d**), and kidney (**e**,**f**), showed varied responses to the treatments administered, but did not necessarily indicate toxicity. Statistical analysis was carried out using a one-way ANOVA test, followed by Tukey’s test for multiple comparisons. Asterisks (*) indicate statistically significant differences with a value of *p* < 0.05. Symbol ‘a’ indicates that the negative control differed significantly from the tested oil concentrations but was equal to the nanoemulsion. Symbol b, oil concentrations tested were statistically different from the NE-OIL in liver weight.

There were no statistically significant differences in the absolute weight of the animals’ spleens between the groups (Figure 12a,b). However, a significant difference was observed in the relative weight of the spleen relative to the total body weight. Specifically, there was a decrease in the relative weight of the spleen in the group treated with the nanoemulsion compared to the negative control group, suggesting a reduction in the spleen’s relative size in the presence of the nanoemulsion (Figure 13a,b).

Regarding the absolute liver weight results (Figure 13c,d), a statistically significant difference was found between the negative control group and the group treated with açai seed oil, with the latter exhibiting a higher liver weight compared to the control. Similarly, a significant difference was observed between the group treated with the oil and the group treated with the nanoemulsion. However, no significant differences were found between the control group and the group treated with the nanoemulsion. Analysis of the relative kidney weight (Figure 13e,f) revealed no statistically significant differences between the groups, indicating that the treatments did not affect the relative size of the liver in relation to body weight. No statistically significant differences were observed in the absolute and relative kidney weight.

In conclusion, the analysis of the absolute and relative organ weights, including the spleen, liver, and kidney, showed varied responses to the treatments administered, but did not necessarily indicate toxicity (Figure 13).

### 3.13. Effect of Açaí Seed Oil and Nanoemulsion on Animal Spleen Cellularity

**Figure 14:**
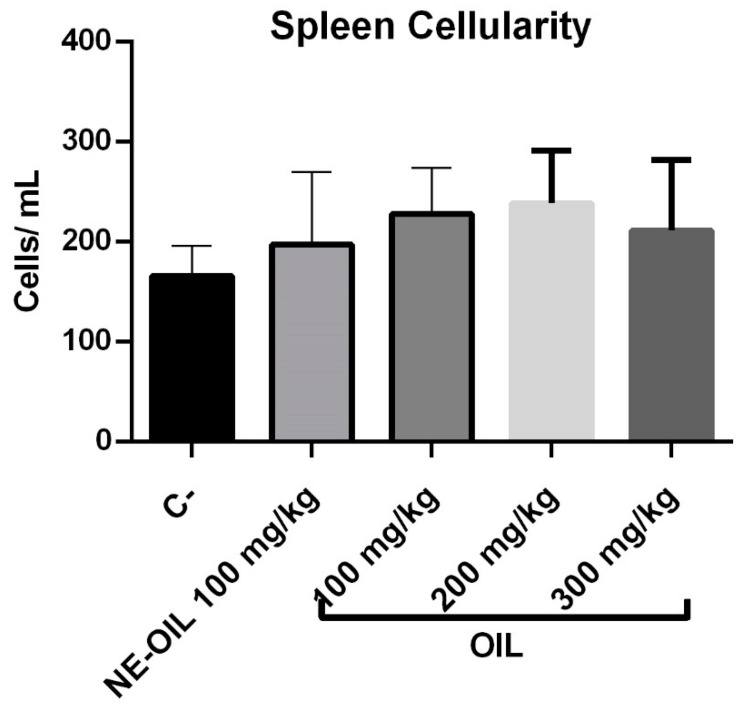
Effect of 100, 200, and 300 mg/kg oil and nanoemulsion (NE-OEO) on animal weight variation at the end of treatment. Statistical analysis was carried out using a one-way ANOVA test, followed by Tukey’s test for multiple comparisons.

Regarding spleen cellularity, there was no statistical difference between açaí seed oil and açaí oil nanoemulsion when compared to control (Figure 14).

### 3.14. Toxicity Analysis in Female Swiss Mice

**Figure 15:**
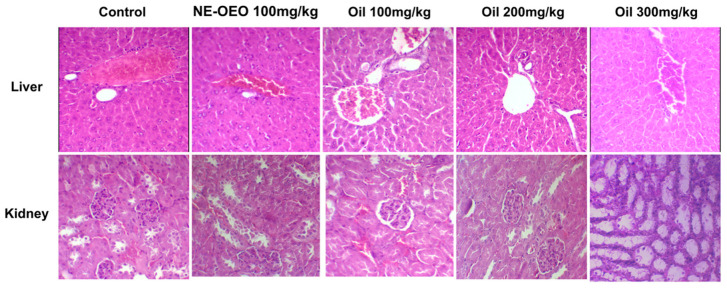
Histological sections of the liver and kidney of female Swiss mice treated with *Euterpe oleracea Mart.* seed oil. Haematoxylin and eosin (HE) staining.

In the histopathological assessment (Figure 15), no significant alterations were observed in the liver and kidneys of most groups, indicating that they were within normal standards compared to the control group.

### 3.15. Açaí Seed Oil and Açaí Seed Nanoemulsion Exerts Immunomodulatory Effects

**Figure 16:**
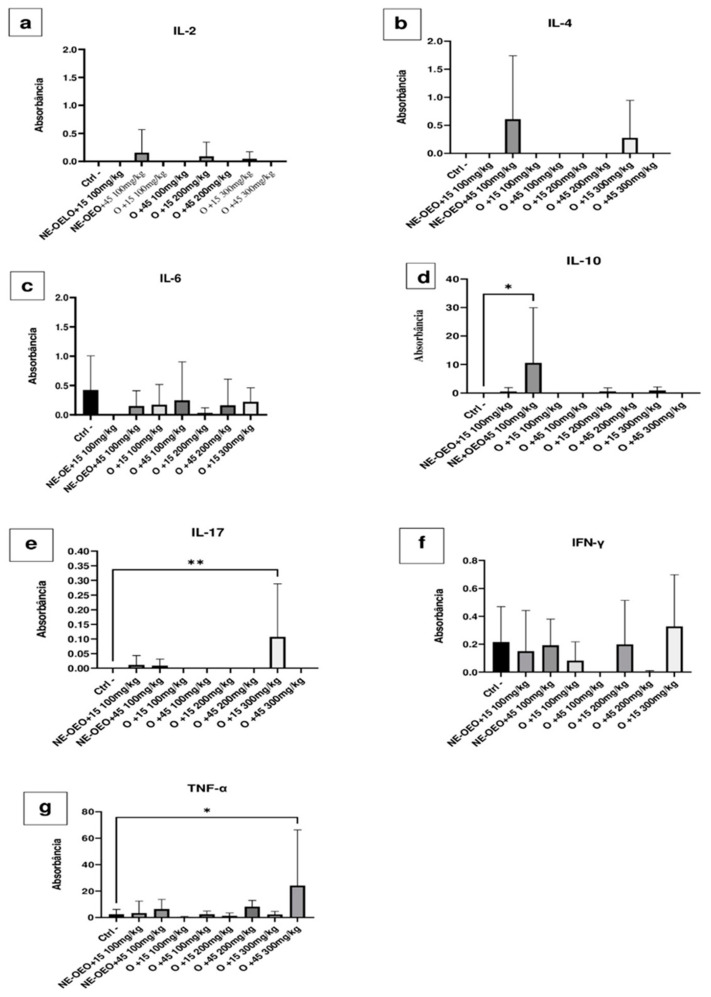
Effect of 100, 200 and 300 mg/kg oil and nanoemulsion (NE-OEO) on the expression of pro-inflammatory and anti-inflammatory cytokines. It is evident that there was a slight induction of IL-2 with NE-OEO at 45 days and 200 mg/kg oil at 15 days (**a**). Similarly, in (**b**), at 45 days, NE-OEO and 300 mg/kg oil induced IL-4. (**c**) reveals a mild expression of IL-6, with NE_OEO at 45 days, 100 mg/kg oil at 15 and 45 days, 200 mg/kg oil at 45 days, and 300 mg/kg oil at 15 days. There was significant expression (*p* < 0.05) of IL-10 (**d**) compared to the control for NE-OEO at 45 days of treatment. (**e**) demonstrates that oil (300 mg/kg) significantly (*p* < 0.05) induced IL-17. NE-OEO and oil discreetly induced IFN-y, with NE-OEO inducing it in both treatment periods, 15 and 45 days, while oil 100 mg/kg, 200 mg/kg, and oil 300 mg/kg only induced it in 15 days (**f**). (**g**) illustrates that 300 mg/mL oil significantly (*p* < 0.05) expressed TNF-a after 45 days of treatment. Statistical analysis was carried out using a one-way ANOVA test, followed by Tukey’s test for multiple comparisons. Asterisks (*) indicate statistically significant differences. * *p* < 0.05, ** *p* < 0.01.

Figure 16 compare the effect of açaí seed oil and NE-OEO on the expression of pro-inflammatory and anti-inflammatory cytokines. 

## 4. Discussion

Seventeen compounds were identified in açaí seed oil (*E. oleracea Mart*.), comprising saturated fatty acids (49.27%), unsaturated fatty acids (50.73%), monounsaturated fatty acids (29.27%), and polyunsaturated fatty acids (20.85%). The predominant esters originated from lauric, myristic, palmitic, linoleic, and oleic fatty acids. These findings are consistent with previous studies by Minighin et al. (2020) [47], reporting similar chemical compositions with saturated fatty acids (23.50%) and monounsaturated fatty acids (63.10%), and studies by Melo et al. (2021), reporting 49.24% saturated and 50.76% unsaturated fatty acids [48].

Although the acidity index (0.3556) and humidity (10% *w*/*w*) exceeded the values stipulated by the National Health Surveillance Agency (RESOLUÇÃO-RDC Nº 270, DE 22 DE SETEMBRO DE 2005) [49] for the acidity category of refined oils (maximum 0.6 mg KOH/g), these findings align with existing literature. Factors such as temperature, atmospheric air, light, fruit ripeness, and humidity may influence these parameters. The slightly elevated humidity percentage may account for the high acidity index observed. This higher water content compromises the product’s durability [50,51].

The saponification index (SI) of the oil (189.61 mg KOH/g) falls within the expected range for vegetable oils and fats according to ANVISA’s standards. The SI reflects the carboxylic group content and the consumption of KOH in the oil sample’s chemical composition, influenced by various factors including species and origin region [50]. The refractive index and density of vegetable oils, physical parameters influenced by fatty acid unsaturation degree, were measured. The obtained values, a refractive index of 1.4707 and a density of 0.928 g/mL, closely align with standard references and previous studies, demonstrating consistency [52]. Regarding ash content, no specific national or international standards exist. However, the measured value of 0.42 g is akin to findings in studies on açaí pulp oil’s fatty acid composition [53]. 

Several studies in the literature have characterised nanoemulsions containing açaí oil, employing diverse methods. Although previous works focused on low-energy methods with different phase inversion temperatures, our study deviated from these approaches [9,54]. Droplet size serves as a key indicator of nanoemulsion stability, with smaller droplets indicating better stability and quality. The particle size distribution’s homogeneity and stability are reflected in the polydispersity index (PDI), where a narrow range (PDI~0.1) signifies monodispersity, while higher values (>0.7) suggest polydispersity, indicative of unstable nanoemulsions [55].

The PDI value indicated the homogeneity and stability of the emulsion’s droplet size distribution (PD. PDI values are usually between 0 and 1. The size distribution of the system is narrow (monodisperse) when the PDI is approximately 0.1 and polydisperse when it is greater than 0.7, which indicates particles with a very broad size distribution that produce unstable nanoemulsions [56]. Zeta potential measurements provide insights into particle stability, with values above 30 mV indicating minimal aggregation due to electrostatic repulsion. The negative zeta potential observed may be attributed to non-ionic surfactants, promoting steric rather than electrostatic stabilisation [57]. Evaluating pH is crucial for monitoring formulation degradation. The neutral pH of the NE-OEO nanoemulsion aligns with previous studies [58]. Turbidity variations reflect component dispersion within the nanoemulsion. NE-OEO’s bluish translucent appearance confirms its nanoemulsion predominance [55].

In assessing antioxidant activity through DPPH and ABTS assays, açaí oil exhibited similar efficacy to previous studies [59]. Effective concentrations below 500 μg/mL indicate significant antioxidant potential, offering health benefits and aiding in oxidative stress-related disease prevention. The dose-dependent relationship between concentration and antioxidant activity highlights the compound’s potency [59].

In cellular assays, 50 μg/mL of açaí oil inhibited cell viability, induced apoptosis, and interfered with migration, invasion, and colony formation in HeLa and SiHa cells. These effects, along with observed morphological changes, underscore the oil’s cytotoxic potential, akin to findings in other essential oil studies [24,25,26,27].

Toxicity assessments revealed no significant alterations in liver and kidney tissues among most animals treated with oil concentrations of 100, 200, and 300 mg/mL, as well as NE-OEO. Conversely, De Marques et al. (2019) reported liver and thyroid cell changes in animals exposed to açaí oil, with increasing doses compromising liver tissue integrity. [60].

## 5. Conclusions

In conclusion, *E. oleracea Mart.* seed oil showed a high content of polyphenols, saturated, unsaturated, monounsaturated, and polyunsaturated acids, with lauric, myristic, palmitic, linoleic, and oleic fatty acids being the majority. The oil also exhibited antitumour activity in vitro against HeLa and Siha cervical cancer cells, inducing changes in cell morphology, inhibiting cell migration and invasion and colony formation and antioxidant activity. In vivo studies did not show any toxicity of açaí oil and açaí nanoemulsion. Overall, açaí seed oil, particularly when formulated into a nanoemulsion, presents potential for cancer treatment due to its bioactive properties and safety profile.

## Figures and Tables

**Table 1:** Content of total phenolic compounds and flavonoids in açaí seed oil and nanoemulsion.

Chemical Quantification	Oil	NE-OEO
Total phenolic content (mg EAG g^−1^) *	127.40 ^a^ ± 0.449	146.00 ^b^ ± 0.259
Total flavonoid content (mg EQ g^−1^) *	62.62 ^a^ ± 0.930	113.80 ^b^ ± 0.454

Mean values of triplicates ± standard deviation/ means followed by different letters on the same line differ statistically (*p* < 0.05) by the unpaired *t*-test. * EAG = gallic acid equivalent. * EQ = quercetin equivalent.

**Table 2:** Physicochemical characterisation of *E. oleracea Mart.* seed oil.

Physicochemical Analysis	Average Values + SD
Acidity (% lauric/oleic acid)	0.3556 ± 0.0003
Misture (*w*/*w*)	1.25 ± 1.64
Saponification index (mg KOH/oil g)	189.61 ± 1.04
Refractive index (40 °C)	1.4707 ± 0.00005
Residue from incineration (g Ash)	0.42 ± 0.42
Density (g/mL)	0.928 ± 0.0005

SD: Standard deviation.

**Table 3:** Non-ionic surfactant mixtures used to obtain the chosen EHL values.

Formulations	Span 80% (*m*/*m*)	Kolliphor^®^ HS 15% (*m*/*m*)	Oil % (*m*/*m*)	Solutions Pbs % (*m*/*m*)	EHL
1	4.5	0.5	10	85	14.83
2	4.0	1.0	10	85	13.66
3	3.5	1.5	10	85	12.49
4	3.0	2.0	10	85	11.32
**5**	**2.5**	**2.5**	10	85	**10.15**
6	2.0	3.0	10	85	8.98
7	1.5	3.5	10	85	7.81
8	1.0	4.0	10	85	6.64
9	0.5	4.5	10	85	5.47

A: Span 80 (EHL 4.3); B: Kolliphor^®^ HS 15 (EHL 16); EHL hydrophilic–lipophilic balance; *m*: mass.

**Table 4:** Analysis of droplet size, polydispersity index (PDI), zeta potential, pH, and turbidity of nanoemulsions prepared with açaí oil after 30 days of storage.

Sample	Drop Size (nm)	PDI	Zeta Potential (mv)	pH	Turbidity (Abs)
NE-OEO	238.37 ± 3.96	0.38 ± 0.38	−9.59 ± 0.11	7.0 ± 0.00.	0.267± 0.00

Average values ± standard deviation (n = 3).

**Table 5:** Trolox-equivalent antioxidant activity of açaí seed oil and nanoemulsion using the DPPH and ABTS methods.

Tests	Antioxidant Activity (µM ET/g) *	
	Oil	NE-OEO
DPPH	5.993 ^a^ ± 0.1925	9.993 ^b^ ± 0.5092
ABTS	6.567 ^a^ ± 0.1667	11.9 ^b^ ± 0.2887

Mean values of triplicates ± standard deviation/ means followed by different letters on the same line differ statistically (*p* < 0.05) by the unpaired *t*-test * ET = Trolox equivalent.

**Table 6:** Antioxidant activity of the 50% effective concentration (EC50) in açaí oil and açaí oil nanoemulsions using the DPPH and ABTS methods.

Tests	EC50 (µg/mL)		
	Oil	NE-OEO	Trolox
DPPH	375,698 ^a^ ± 9054	229,845 ^b^ ± 10,680	10,132 ^c^ ± 0.00
ABTS	272,0208 ^a^ ± 9913	201,2895 ^b^ ± 9849	4053 ^b^ ± 0.00

Mean values of triplicates ± standard deviation/ means followed by different letters on the same line differ statistically (*p* < 0.05) by the unpaired *t*-test. Source: authors, (2023).

**Table 7:** Morphological analysis of HeLa and SiHa cells after treatment with *E. oleracea Mart*. seed oil.

Cell Morphology	Control		Treatment			
	HeLa	SiHa	HeLa 24 h	HeLa 48 h	Siha 24 h	Siha 48 h
Area	53.8 ± 30.0 µm^2^	72.2 ± 16.5 µm^2^	430.6 ± 14.4 µm^2^	60.6 ± 19.7 µm^2^	220.6 ± 89.24 µm^2^	60.35 ± 29.9 µm^2^
Perimeter	28.0 ± 9.9 µm^2^	33.7 ± 5.9 µm^2^	72.7 ± 11.9 µm^2^	27.4 ± 4.7 µm^2^	57.57 ± 19.96 µm^2^	27.0 ± 6.2 µm^2^

## Data Availability

All data are included in the manuscript.
